# Supplementary material for: A Mindfulness-Based Mobile Application’s Impact on Nurse Burnout Syndrome and Well-Being
Source: Healthcare (Basel). 2025 Sep 23;13(19):2386. doi: 10.3390/healthcare13192386 (PMC12523357; doi:10.3390/healthcare13192386)
Supplement: Supplementary file 1 [file healthcare-13-02386-s001.zip › healthcare-3817991-supplementary.pdf]

Included Analyses

- Pearson Correlation Analysis for Pre_MZSI and Pre_NWBI

Results

Pearson Correlation Analysis

Introduction

A Pearson correlation analysis was conducted between Pre_MZSI and Pre_NWBI. Cohen's standard was used to evaluate the strength of the relationship, where coefficients between .10 and .29 represent a small effect size, coefficients between .30 and .49 represent a moderate effect size, and coefficients above .50 indicate a large effect size (Cohen, 1988).

Assumptions

Linearity. A Pearson correlation requires that the relationship between each pair of variables is linear (Conover & Iman, 1981). This assumption is violated if there is curvature among the points on the scatterplot between any pair of variables. Figure S1 presents the scatterplot of the correlation. A regression line has been added to assist the interpretation.

Figure S1. *Scatterplots with the regression line added for Pre_MZSI and Pre_NWBI*

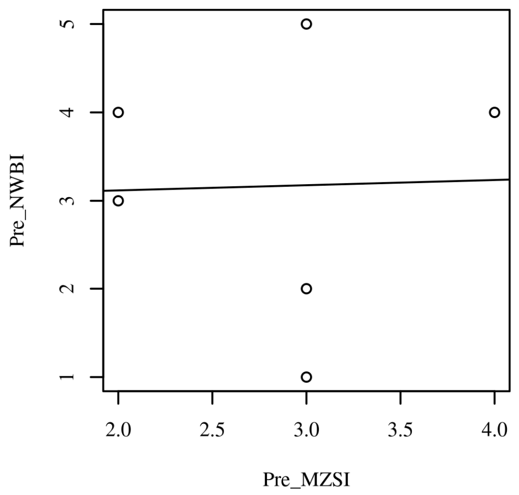

Results

The result of the correlation was examined based on an alpha value of .10. There were no significant correlations between any pairs of variables. Table S1 and Table S2 presents the results of the correlation.

Table S1. *Pearson Correlation Matrix Between Pre_MZSI and Pre_NWBI*

Variable	1	2
1. Pre_MZSI	-	
2. Pre_NWBI	0.03	-

Table S2. *Pearson Correlation Results Between Pre_MZSI and Pre_NWBI*

Combination	*r*	90.00% CI	*n*	*p*
Pre_MZSI-Pre_NWBI	0.03	[-0.73, 0.75]	6	0.955

References

Cohen, J. (1988). *Statistical power analysis for the behavior sciences* (2nd ed.). West Publishing Company.

Conover, W. J., & Iman, R. L. (1981). Rank transformations as a bridge between parametric and nonparametric statistics. *The American Statistician, 35*(3), 124-129. https://doi.org/10.1080/00031305.1981.10479327

Intellectus Statistics [Online computer software]. (2025). Intellectus Statistics. https://statistics.intellectus360.com

Glossaries

Pearson (Product-Moment) Correlation

A correlation expresses the strength of linkage or co-occurrence between two variables in a single value between -1 and +1. This value that measures the strength of linkage is called *correlation coefficient*, which is represented typically as the letter r. The correlation coefficient between two continuous-level variables is also called Pearson's r or Pearson product-moment correlation coefficient. A positive r value expresses a positive relationship between the two variables (the larger A becomes, the larger B becomes) while a negative r value indicates a negative relationship (the larger A becomes, the smaller B becomes). A correlation coefficient of

zero indicates no relationship between the variables. However, correlations are limited to linear relationships between variables. Even if the correlation coefficient is zero, a non-linear relationship might exist.

Fun Fact! *Correlation is a widely used term in statistics. In fact, it entered the English language in 1561, 200 years before most of the modern statistic tests were discovered. It is derived from the [same] Latin word correlation, which means relation.*

Bonferroni Correction: If one conducts a lot of correlations, some relationships will occur by chance. To mitigate this, Bonferroni correction is applied. It reduces the alpha level for the analysis, thus reducing the likelihood of making a Type I error (false positive); it is based on the number of times each variable is used.

Correlation Coefficient (*r*): Ranges from -1 to 1; describes to the strength of the relationship between the variables.

Critical Value: The minimum value at which an observed correlation coefficient is statistically significant.

***p*-value:** The probability of obtaining the observed results if the null hypothesis is true. A result is usually considered statistically significant if the *p*-value is ≤ .05.

Raw Output

Pearson Correlation Test

Included Variables:
Pre_MZSI and Pre_NWBI

Sample Size (Complete Cases):
N = 6

Correlation Matrix:

Variable	1	2
1. Pre_MZSI	-	
2. Pre_NWBI	0.0301	-

Note. '*' indicates p < 0.100.

Correlation Results:

Combination	r	90.000% CI	n	p
Pre_MZSI-Pre_NWBI	0.0301	[-0.726, 0.753]	6	0.955
